# Pigments in Extra-Virgin Olive Oils Produced in Tuscany (Italy) in Different Years

**DOI:** 10.3390/foods6040025

**Published:** 2017-03-29

**Authors:** Cristina Lazzerini, Valentina Domenici

**Affiliations:** Dipartimento di Chimica e Chimica Industriale, Università di Pisa, 56124 Pisa, Italy; lazzerinicristina@gmail.com

**Keywords:** olive oils, carotenoid, chlorophyll, ultraviolet-visible spectroscopy, multivariate analysis, β-carotene, lutein, pheophytin A, Tuscany

## Abstract

Pigments are responsible for the color of olive oils, and are an important ingredient that is directly related to the quality of this food. However, the concentration of pigments can vary significantly depending on the climate conditions, harvesting time, and olive cultivars. In this work, we quantified the main pigments in several extra-virgin olive oils produced from a blend of three cultivars (*Moraiolo*, *Frantoio*, and *Leccino*) typical of Tuscany (Italy) harvested in three different years: 2012, 2013, and 2014. Pigments—namely, β-carotene, lutein, pheophytin A, and pheophytin B—were quantified by a method based on the mathematical analysis of the near ultraviolet-visible absorption spectra of the oils. Data were analyzed by a multivariate statistical approach. The results show that the pigments’ content of extra-virgin olive oils produced in 2014 can be well distinguished with respect to previous years. This can be explained by the anomalous climate conditions, which strongly affected Italy and, in particular, Tuscany, where the olives were harvested. This study represents an interesting example of how pigment content can be significant in characterizing olive oils. Moreover, this is the first report of pigment quantification in extra-virgin olive oils produced in Tuscany.

## 1. Introduction

Extra-virgin olive oil is an essential food in Mediterranean cuisine, and is nowadays an appreciated and recognized ingredient in many other cultures. Olive oil is produced from the olive fruits of the *Olea europeae* L. trees, and due to the high content of monounsaturated fatty acids and bioactive compounds [1], olive oil is considered beneficial for human health [2]. Among various types of olive oils, virgin olive oils (VOOs) and extra-virgin olive oils (EVOOs) are those with the highest content of minor compounds having bioactive properties (about 1%–2%). They are divided into polar phenols and their derivatives, and non-polar (unsaponifiable) compounds, such as squalene and other triterpenes, sterols, tocopherols, and pigments [1,2]. Pigments in olive oils can be divided in two main classes: carotenoids and chlorophyll derivatives [3,4,5]; they are responsible for the color of olive oils [6], which is an important feature for the quality of EVOO. Moreover, pigments’ bioactivity is associated with their healthy properties for several human organs, such as the brain and nervous system [7,8]. 

Olive oils contain a relatively rich variety of carotenoids (i.e., β-carotene, lutein, violaxanthin, neoxanthin, and other xanthophylls) and chlorophyll derivatives (i.e., chlorophylls A and B, pheophytins A and B, and other minor derivatives) [3]. Their relative composition in olive oil derives from the initial pigment composition of the olive fruits and from all chemical transformations, such as those which are enzymatically driven, occurring at different stages of olive oil production [4]. The main factors affecting the pigment profile of olive fruits are the variety (or cultivar) [3,9,10,11,12], the ripening degree [13,14,15], the climate [16,17], and growing [18] conditions. Further influence on the final content and percentage of pigments in olive oil is from the specific conditions of oil production, such as malaxation stage and oil extraction [19,20,21].

Most papers reporting on pigment content in olive oils provide only the total amount of carotenoids and chlorophyll derivatives, determined by measuring the absorbance of olive oil dissolved in cyclohexane [6], or of olive oil directly as it is [22]. On the contrary, the identification and quantification of single pigments is usually done by the use of chromatographic methods, such as high performance liquid chromatographic (HPLC) with ultraviolet-visible (UV-vis) detection [23]. Recently, a new method based on the quantitative analysis of the absorption spectrum of olive oil in the near UV-vis range has been developed [24], able to determine the concentration of four main pigments: β-carotene and lutein among the carotenoids, and pheophytin A and pheophytin B among chlorophyll derivatives. This method has been applied to extra-virgin olive oil samples produced in several Mediterranean countries from different cultivars [5,14,25,26,27], confirming its validity, goodness, and high reproducibility in the quantification of the four main pigments present in olive oil. 

In this work, we focus on the pigment content of extra virgin olive oil samples produced in Tuscany (Italy) in different harvesting years. The region of Tuscany (on the western coast of central Italy) is among Italy’s leading olive oil producers, and EVOOs produced in this region—which represent about the 2%–4% of total Italian EVOO production [28,29]—are considered to be of very high quality. The main olive cultivars typical of Tuscany are *Frantoio*, *Moraiolo*, and *Leccino* [29]—each of them with characteristic properties. *Frantoio* is characterized by large fruits, *Frantoio* trees have high and constant productivity, good adaptation capacity, and weather-resistance; the organoleptic properties of olive oil produced by *Frantoio* are considered excellent. *Moraiolo* has a medium–low productivity, but high yields; it has a low resistance against different plant diseases, but it is resistant to windy and hot weather [29]. *Leccino* is very resistant to different weather conditions (cold, wind, temperature gaps, etc.), but has low resistance to fruit drop [28,29]. *Leccino* is sterile and needs to be pollinated, typically from *Frantoio* plants. In Tuscany, for both traditional and botanical reasons, monocultivar olive oil production is rare, while most high-quality Tuscan EVOOs (such as *identificazione geografica protetta*, IGP, and *denominazione di origine protetta*, DOP, labeled EVOOs) are obtained from a blend of these three varieties. The scientific interest in these cultivars is demonstrated by numerous studies, mainly dealing with the phenolic profiles and antioxidant properties [17,30,31], as well as sensorial and organoleptic features [32,33,34] of Tuscan EVOOs. Several works have investigated the effect of the ripening period and climate conditions [31,35], olive processing and extraction methods [36], and the effect of different storage conditions [34] on extra-virgin and virgin olive oils produced by these three Tuscan cultivars. So far, no works have been reported concerning the pigment content in extra virgin olive oils produced in Tuscany from the main cultivars *Frantoio*, *Moraiolo*, and *Leccino*. 

In the present work, the pigment profile of a selection of EVOOs produced from a blend of these three varieties in several Tuscan growing areas and obtained in three different years (i.e., 2012, 2013, and 2014) was determined by analyzing their near ultraviolet–visible absorption spectra. Results are analyzed by a multivariate approach based on principal component analysis (PCA) and discussed taking into account the peculiarity of the 2014 crop season, which was characterized by one of the strongest olive fruit fly attacks in Tuscany [37]. 

## 2. Materials and Methods 

### 2.1. Samples

**Figure 1:**
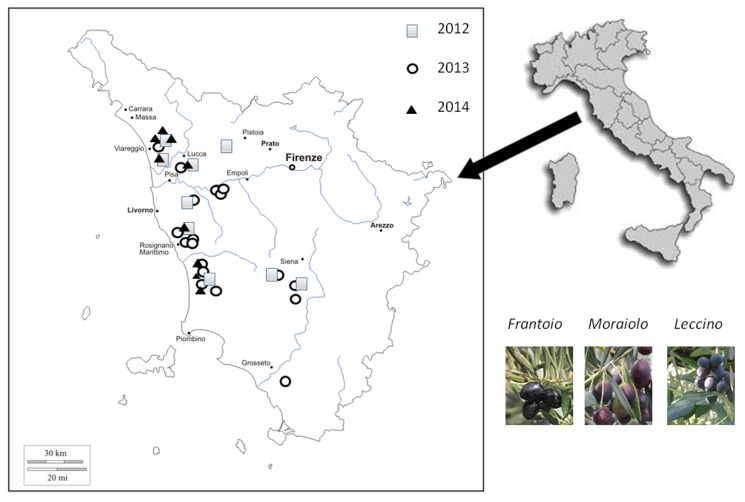
Extra-virgin olive oil samples of different harvesting years (2012, 2013, and 2014) produced in Tuscany (Italy) from a blend of *Leccino*, *Moraiolo*, and *Frantoio* cultivars. The location of olives’ origin is indicated for all investigated samples.

Several EVOO samples produced in Tuscany (Italy) from *Frantoio*, *Moraiolo*, and *Leccino* varieties were provided by private producer companies. These samples were obtained from olives (fruits of *Olea europaea*, L. trees) harvested in three different seasons (2012, 2013, and 2014). In the case of 2012 samples, harvesting was performed from the 23rd of October to the 9th of November; 2013 samples were harvested from the 18th of October until the 11th of November; in the case of 2014 samples, harvesting was limited to the period from the 16th to the 28th of October. For all harvesting periods, the climate conditions are known and they can be found on the web [38,39]. The local areas of olive trees growing and olive harvesting are reported in Figure 1. Most of the samples were produced in areas close to the seaside (districts of Lucca, Livorno, Pisa, and Grosseto), and only few samples were produced in more inland areas (districts of Pistoia and Siena). These samples were labelled from T1 to T37. In particular, we had nine samples from the 2012 harvest (from T1 to T9), eighteen samples from the 2013 harvest (from T10 to T27), and ten samples from the 2014 harvest (from T28 to T37). The ripeness stage of olives at harvesting was purple (P)/black (B), according to the ripeness index defined by Loudiyi et al. [40]. All samples were classified as “extra virgin olive oils” by sensory characteristics (International Regulations, Reg. CE 640/2008) and analytical indices (European Regulation, Reg. CE 1234/2007, annex XVI). All EVOO samples were stored in dark glass bottles in the dark at 5 °C, and the chemical–physical analysis was performed three months after olive oil production [41].

### 2.2. Method

Near UV-visible absorbance spectra of extra virgin olive oil samples were measured with a Jasco V-550 spectrophotometer using quartz cells with 0.2 cm optical path length [24,25]. Before absorbance measurements, the oil samples were centrifuged for 30 min at 5000 rpm in order to minimize the light absorption and scattering phenomena due to suspended particles. The absorbances of EVOO spectra were than referred to a 1 cm optical path and analysed by using a mathematical tool compatible with Excel, developed by Domenici et al. [24].

**Figure 2:**
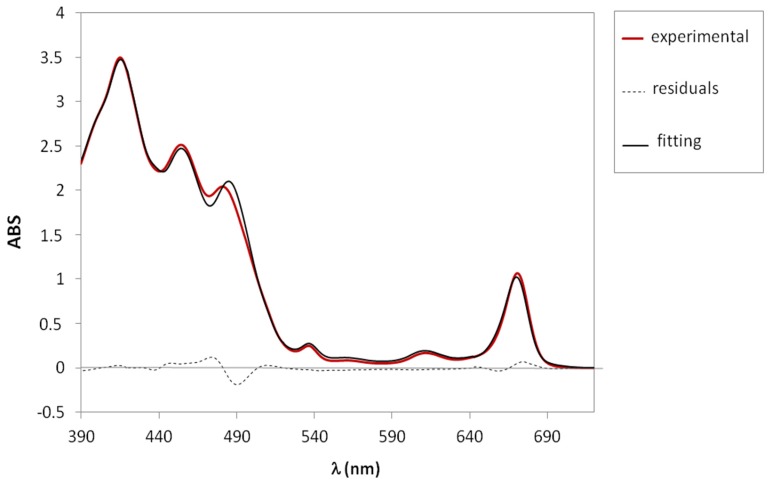
Example of near ultraviolet-visible (UV-vis) absorption spectrum of an extra-virgin olive oil (EVOO) sample, recorded in the range 390–720 nm. Experimental (red) and fitted (black) curves are reported with the residuals (dotted) curve.

The mathematical treatment of the EVOO absorption spectrum allowed us to extract the concentration of the four main pigments (β-carotene, lutein, pheophytin A, and pheophytin B). This mathematical approach consists of the deconvolution of the experimental spectrum in terms of four orthogonal spectra obtained from the original experimental spectra of the four pigments, as described in detail in Reference [24]. This fitting procedure gives us as an output file the concentration of the four pigments and other relevant parameters such as the ratio between the total amount of carotenoids and chlorophyll derivatives, the percentage of lutein with respect to the carotenoid fraction, and so on. As an example, the experimental spectrum of an EVOO sample analysed in this work is reported in Figure 2 (red curve) together with the fitted spectrum (black curve). The residuals can also be visualized as a dotted curve. The goodness of the mathematical treatment can be verified by the “*R*-square” test (*R*^2^), which estimates the correlation between the experimental values and the values predicted by the deconvolution procedure, and defined as:

(1) $$R-square=1-\frac{\sum_{i=1}^{n}{\left(y_{i}-f_{i}\right)}^{2}}{\sum_{i=1}^{n}{\left(y_{i}-\bar{y}\right)}^{2}}$$

where *f_i_* is the value predicted by the fitting, $\bar{y}$ is the mean of the observed data, and *y_i_* is the observed data value (i.e., the absorbance value at a certain (*i*) wavelength).

As previously reported [24,25,26,27,41], this method was shown to be robust with high reproducibility and good sensitivity. For each EVOO sample analysed in this work, the near UV-visible spectra were measured with three replicates, and the values of concentration of the four pigments are expressed as average value ± standard deviation (over three replicates). The coefficient *R*^2^ of the fitting is reported for each sample in order to evaluate the goodness of the mathematical treatment.

In previous works [14,25,26], we have tested the UV-vis method by applying it to a series of samples with known main pigments’ concentrations, and the method was shown to be accurate. The only limitation—which is not addressed in this work—is related to the eventual presence of other minor pigments (e.g., other xanthophylls), which may contribute to the absorption in the region of the spectrum around 490 nm, which corresponds to the highest residuals in absolute values, as shown in Figure 2. 

### 2.3. Statistical Analysis

Univariate and multivariate analyses were performed by using XLSTAT software (Addinsoft, Paris, France) for EXCEL. 

## 3. Results and Discussion

### 3.1. Pigments Quantification

**Figure 3:**
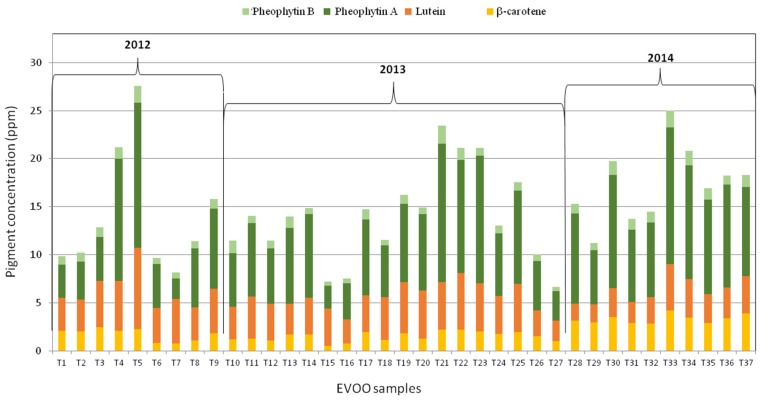
Profile of the four main pigments (β-carotene, lutein, pheophytin A, and pheophytin B) determined by the mathematical analysis of the near UV-vis absorption spectra of the EVOO samples investigated in this work. Samples are labelled from T1 to T37, as shown in Table 1. Three groups of samples can be visualized depending on the year of olive harvesting (2012, 2013, and 2014).

The main pigments—two carotenoids and two chlorophyll derivatives—were quantified by analyzing the near UV-visible spectrum of EVOO samples produced in three consecutive harvesting years from 2012 to 2014, obtained from olives produced in the western part of Central Italy, namely in Tuscany (Figure 1), from three typical cultivars: *Leccino*, *Moraiolo*, and *Frantoio*. The concentration (ppm) of β-carotene, lutein, pheophytin A, and pheophytin B of the EVOO samples, labeled from T1 to T37, are reported in Table 1 and in Figure 3. 

The concentrations of the four pigments were obtained with relatively high accuracy [14,25,26] in comparison with conventional chromatographic methods [3,4,5,6]. The goodness of the fitting procedure needed for the extraction of pigment contents from the analysis of the absorption spectrum was confirmed by the values of the determination coefficient, *R*^2^, which reached the highest value of 0.999 for three samples (T4, T14, and T26) and the lowest value of 0.983 for one sample (T32). 

In Figure 3, the variation of pigment content among the EVOO samples can be well visualized. As a general remark, the highest content of pigments was obtained for an EVOO sample produced in 2012 (27.58 ppm), and the lowest value of total pigments was 6.65 ppm, obtained in an EVOO sample produced in 2013. The average value of the total amount of pigments observed in this set of EVOO samples was about 15 ppm, but—as seen from the pigment profile shown in Figure 3—the differences related to the total amount of pigments among the three years are quite significant, without showing specific trends within each year. 

However, there are other parameters related to the pigments’ content which vary significantly among the three years of olive harvesting. An important parameter associated with pigments is the ratio between total amount of chlorophyll derivatives and the total amount of carotenoids—namely, the ratio P/C. The average value of P/C in the three years was: 1.18 ± 0.46 (2012), 1.46 ± 0.37 (2013), and 1.73 ± 0.26 (2014). This ratio is considered a quality parameter for EVOO samples [4,5]. For instance, according to Roca and Minguez-Mosquera [42], this ratio should be around 1.14, shifting in the range 0.53–1.40 depending on the quality of EVOOs. Other studies indicate that this ratio can assume values in a much larger range, because it may vary sensibly depending on the cultivar, geographic olive trees cultivation, and climate conditions [9,12,13,14,26], as confirmed by the case presented in this work. 

**Figure 4:**
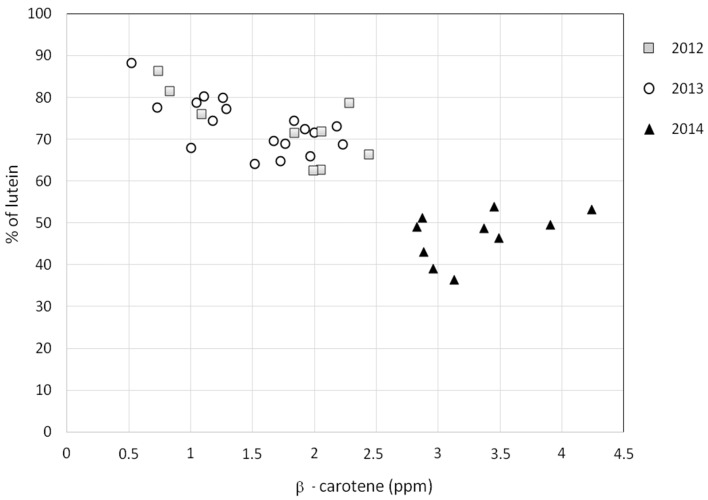
Plot of the parameter “% of lutein” (over the carotenoid fraction) versus the concentration (ppm) of β-carotene for the EVOO samples investigated in this work. Samples are labeled according to the harvest year.

Another important parameter is the percentage of lutein over the sum of carotenoids (% of lutein) [4,5]. In this respect, the differences between the first two years (2012 and 2013) and the year 2014 were very significant. The average value of “% of lutein” was 73% ± 8% and 73% ± 6% in EVOOs produced in 2012 and in 2013, respectively. In the case of EVOOs produced in 2014, this percentage was much lower (47% ± 6%). As shown in Figure 4, the anomaly of the EVOO samples produced in 2014 is related to the high content of β-carotene with respect to lutein. 

### 3.2. Multivariate Data Analysis

**Figure 5:**
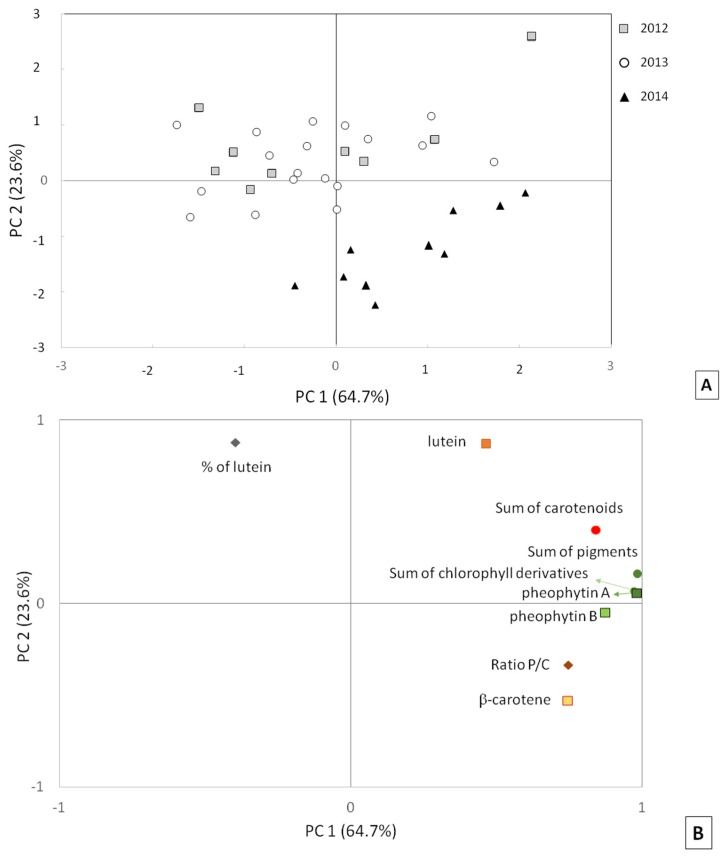
Results of the multivariate analysis. (**A**) Score plot of the principal component analysis (PCA) modelling, showing the EVOO samples harvested in 2012 (■), 2013 (**○**), and 2014 (▲); (**B**) Loading plot of the PCA modelling, showing the variables used for the analysis. P/C: ratio between total amount of chlorophyll derivatives and the total amount of carotenoids.

Data obtained by the analysis of near UV-visible absorption spectra were further analyzed by means of principal component analysis (PCA). In this analysis, we have identified nine variables: the concentrations of the four pigments, the total amount of pigments, the carotenoid and chlorophyll derivative fractions, the P/C ratio, and the % of lutein. The PCA results reported in Figure 5 indicate that the parameters analyzed have a distinct response pattern in EVOOs produced in 2012 and 2013 compared to EVOOs produced in 2014, supporting previous considerations. In particular, PCA analysis produced two significant components that, together, explain 88.3% of the total variance. The first component (PC 1, explaining 64.7% of the total variance) includes seven variables (β-carotene, pheophytins A and B, sum of pigments, total carotenoids, total chlorophyll derivatives, and the P/C ratio). Lutein and the % of lutein are associated with the second component PC 2 (23.6% of the variance).

A scatter plot of the scores of different treated samples in the score space PC 1/PC 2 is shown in Figure 5A. EVOO samples produced in 2014 (black triangles) are clearly distinguished from the EVOOs produced in 2012 (grey squares) and 2013 (empty circles). In particular, almost all 2014 EVOO samples are in the fourth quarter (positive PC 1 and negative PC 2), while 77% of EVOO samples produced in 2012 and 2013 have positive PC 2 (first and second quarters).

The PCA loading plot is reported in Figure 5B, showing the distribution of variables. Some of them are highly correlated, such as the sum of pigments, the sum of chlorophyll derivatives, and the single concentrations of pheophytins A and B. Their combined use in the multivariate analysis does not modify the results. All other parameters are not significantly correlated. 

The PCA results confirm previous observations about the anomalous values of β-carotene and % of lutein of samples produced in 2014 with respect to previous years. These anomalies can be associated with the particular conditions of the 2014 crop season. As reported in Reference [37], the climatic anomalies observed during the period from June to October 2014 [38,39] strongly affected Tuscany, and almost all olive trees were damaged by the attack of *Bactrocera oleae*. The olive oils produced in that period in Tuscany were characterized by lower values of phenolic/tocopherol compounds and higher free acidity than previous harvesting years. The fly attack brought to an alteration of the metabolic processes typical of the olives [37] and a different exposure to oxygen. These reasons may be at the origin of an alteration of the pigment profile observed in the present study. Other reasons could be related to the different harvesting period, which was slightly early in 2014 with respect to 2012 and 2013. The anticipation of harvesting on the other hand was related to the particular weather conditions. Other factors usually influencing olive oil pigment profiles, such as cultivar, geographic origin, olive oil treatment and production processes seem not be relevant in this case, since all these factors were the same in the three different harvesting years. 

## 4. Conclusions

Pigment content of extra virgin olive oils produced from a blend of three cultivars typical of Tuscany—namely *Leccino*, *Frantoio*, and *Moraiolo*—in three different years (2012, 2013, and 2014) was determined by analyzing the absorption spectrum in the near UV–visible region of each sample. The method is fast, non-destructive, and relatively cheap, and allowed us to obtain the concentration of the four main pigments present in EVOOs: β-carotene, lutein, pheophytin A, and pheophytin B. Relevant parameters, such as the ratio between the chlorophyll derivatives and the carotenoids (P/C) and the percentage of lutein (% lutein) were calculated. The results show that EVOOs produced in 2014 had a high content of β-carotene with respect to lutein, showing an anomalous value of % of lutein. The anomalous climate conditions affecting Tuscany between June and October 2014—which in turn were at the origin of one of the strongest fly attack of the last decade—are among the most probable reasons of the anomalous pigment profile in EVOO produced in 2014, with respect to previous years. In fact, all other factors influencing the pigment profiles in olive oil were the same in the three harvesting years. The anomalous climate conditions and the consequent fly attack could have affected the metabolism of olives and olive oils, resulting in a different pigment profile. This hypothesis is in agreement with other studies showing that olive oils produced in Tuscany in 2014 had anomalous content in phenolic/tocopherol compounds, free acidity, and other chemical properties. Multivariate analysis demonstrated that EVOO samples produced in Tuscany from a blend of three typical cultivars, namely *Leccino*, *Moraiolo*, and *Frantoio*, in 2014 can indeed be differentiated from previous harvesting years (i.e., 2012 and 2013) by their pigment composition and most relevant parameters.

## Figures and Tables

**Table 1:** Concentration (ppm) of the four main pigments in EVOO samples, labeled from T1 to T37, from *Leccino*, *Moraiolo*, and *Frantoio* cultivars produced in different years in Tuscany. Data are obtained from the analysis of the near UV-vis absorption spectra. The concentration of pigments is expressed as average value ± standard deviation (three replicates each). The coefficient *R*^2^ of the fitting is reported for each sample.

EVOO Label	β-Carotene (ppm)	Lutein (ppm)	Pheophytin A (ppm)	Pheophytin B (ppm)	*R*^2^
**From 2012 Harvesting**					
T1	2.05 ± 0.19	3.46 ± 0.15	3.52 ± 0.11	0.87 ± 0.03	0.994
T2	1.99 ± 0.20	3.32 ± 0.31	4.03 ± 0.13	0.94 ± 0.03	0.997
T3	2.44 ± 0.18	4.82 ± 0.25	4.62 ± 0.16	1.00 ± 0.05	0.998
T4	2.05 ± 0.15	5.24 ± 0.61	12.67 ± 0.13	1.23 ± 0.04	0.999
T5	2.28 ± 0.11	8.47 ± 0.10	15.08 ± 0.17	1.73 ± 0.02	0.998
T6	0.83 ± 0.18	3.64 ± 0.82	4.63 ± 0.15	0.64 ± 0.05	0.998
T7	0.73 ± 0.15	4.67 ± 0.22	2.14 ± 0.22	0.62 ± 0.02	0.998
T8	1.08 ± 0.12	3.46 ± 0.11	6.11 ± 0.13	0.78 ± 0.05	0.998
T9	1.83 ± 0.11	4.63 ± 0.14	8.33 ± 0.18	0.98 ± 0.06	0.998
**From 2013 Harvesting**					
T10	1.17 ± 0.12	3.42 ± 0.12	5.53 ± 0.12	1.36 ± 0.03	0.997
T11	1.29 ± 0.11	4.37 ± 0.08	7.64 ± 0.22	0.74 ± 0.05	0.998
T12	1.04 ± 0.18	3.86 ± 0.13	5.78 ± 0.16	0.81 ± 0.06	0.998
T13	1.73 ± 0.14	3.17 ± 0.21	7.83 ± 0.23	1.20 ± 0.10	0.998
T14	1.67 ± 0.11	3.83 ± 0.13	8.68 ± 0.29	0.66 ± 0.08	0.999
T15	0.52 ± 0.21	3.88 ± 0.41	2.41 ± 0.10	0.42 ± 0.05	0.998
T16	0.73 ± 0.15	2.52 ± 0.03	3.74 ± 0.12	0.50 ± 0.06	0.998
T17	1.96 ± 0.18	3.81 ± 0.22	7.93 ± 0.11	1.06 ± 0.03	0.998
T18	1.10 ± 0.18	4.47 ± 0.12	5.43 ± 0.11	0.55 ± 0.08	0.998
T19	1.83 ± 0.13	5.32 ± 0.21	8.12 ± 0.10	0.91 ± 0.03	0.998
T20	1.26 ± 0.10	5.03 ± 0.31	8.05 ± 0.15	0.66 ± 0.15	0.998
T21	2.23 ± 0.09	4.89 ± 0.43	14.41 ± 0.18	1.89 ± 0.06	0.998
T22	2.18 ± 0.12	5.93 ± 0.46	11.68 ± 0.21	1.28 ± 0.03	0.997
T23	2.00 ± 0.18	5.04 ± 0.10	13.34 ± 0.11	0.86 ± 0.02	0.998
T24	1.76 ± 0.14	3.92 ± 0.12	6.57 ± 0.15	0.76 ± 0.01	0.998
T25	1.92 ± 0.14	5.05 ± 0.12	9.73 ± 0.10	0.91 ± 0.03	0.997
T26	1.51 ± 0.15	2.70 ± 0.14	5.12 ± 0.18	0.69 ± 0.04	0.999
T27	1.00 ± 0.21	2.12 ± 0.15	3.10 ± 0.16	0.43 ± 0.06	0.998
**From 2014 Harvesting**					
T28	3.12 ± 0.14	1.79 ± 0.18	9.32 ± 0.13	1.02 ± 0.02	0.993
T29	2.95 ± 0.19	1.90 ± 0.22	5.64 ± 0.16	0.77 ± 0.05	0.991
T30	3.49 ± 0.18	3.03 ± 0.12	11.78 ± 0.11	1.44 ± 0.03	0.988
T31	2.88 ± 0.22	2.18 ± 0.25	7.65 ± 0.10	1.09 ± 0.04	0.996
T32	2.82 ± 0.12	2.73 ± 0.10	7.83 ± 0.09	1.13 ± 0.08	0.983
T33	4.23 ± 0.26	4.82 ± 0.21	14.22 ± 0.11	1.79 ± 0.03	0.989
T34	3.45 ± 0.28	4.03 ± 0.26	11.77 ± 0.15	1.49 ± 0.00	0.997
T35	2.87 ± 0.26	3.01 ± 0.18	9.94 ± 0.18	1.20 ± 0.02	0.991
T36	3.37 ± 0.09	3.20 ± 0.21	10.74 ± 0.21	0.92 ± 0.02	0.998
T37	3.90 ± 0.14	3.85 ± 0.17	9.31 ± 0.10	1.29 ± 0.05	0.998
